# Assessment of Performance in Youth Soccer Players: Should We Consider the Maturation Status?

**DOI:** 10.5114/jhk/184276

**Published:** 2024-05-17

**Authors:** Fabrizio Perroni, Stefano Amatori, Lorenzo Corsi, Roberto Bensi, Laura Guidetti, Carlo Baldari, Marco B. L. Rocchi, Carlo Castagna, Erica Gobbi, Davide Sisti, Cosme Franklim Buzzachera

**Affiliations:** 1 Department of Biomolecular Sciences, Section of Exercise and Health Sciences, University of Urbino Carlo Bo, Urbino, Italy.; 2 “Museum of Football F.I.G.C.” Foundation, Italian Football Federation, Rome, Italy.; 3 Fitness Training Area, Italian Referee Association of Italian Football Federation, Rome, Italy.; 4 Faculty of Psychology, eCampus University, Novedrate, Como, Italy.; 5 Unicusano Department, University “Niccolò Cusano”, Rome, Italy.; 6 Department of Biomolecular Sciences, Service of Biostatistics, University of Urbino Carlo Bo, Urbino, Italy.; 7 Fitness Training and Biomechanics Laboratory, Technical Department of the Italian Football Federation, Coverciano, Florence, Italy.; 8 Department of Public Health, Experimental and Forensic Medicine, University of Pavia, Pavia, Italy.

**Keywords:** chronological age, football, pubertal development, relative age, talent identification

## Abstract

The influence of biological maturity status on talent identification and development in youth soccer has been debated extensively. Alternative methods have thus recently emerged to estimate maturity status, such as the Pubertal Development Scale (PDS), but their relationship with physical capabilities of young soccer players still needs to be determined. The present study investigated the relationships of different PDS-derived pubertal status measures, chronological age, and relative age with selected performance variables in youth soccer. Sixty-one male soccer players were assessed for physical capabilities using field tests for sprinting, vertical jumps (countermovement jump, CMJ), intermittent high-intensity endurance, and repeated sprint ability. Chronological age was defined as the number of days since birth, and relative age was defined in terms of age quarters. PDS-derived measures of puberal status, otherwise, were determined as an average PDS score, a PDS category score, and a pubertal category. Chronological age, relative age, and measures of pubertal status were scarcely related (p > 0.05) to selected measures of soccer performance. Significant correlations were only found between different measures of pubertal status and the variable “work” in the CMJ test (range r = 0.33–0.36; p < 0.01) and between chronological age and CMJ height (r = −0.297; p = 0.02). The present results suggest that physical performance of young soccer players is poorly related to chronological age, relative age, and pubertal status. Potential effects of biological maturity status on physical capabilities may not be easily identifiable in a group of young soccer players narrowed in terms of chronological age and training status.

## Introduction

Talent identification refers to a complex process that arises from multiple factors that operate in various ways to identify individuals who have the potential to become elite players (Williams and Reilly, 2000). Those recognized as talented are given an invitation to join development programs where they can access a suitable learning environment that will enable them to fully develop their potential (Reilly et al., 2000). Both processes of identifying and developing young talents have become topics of interest in modern soccer (Carling et al., 2009). Evidence-based support systems, that take a comprehensive approach for talent identification and development, have emerged and are gradually replacing the dominant, non-scientific model of identifying promising youth soccer players based on the coach's subjective preconceived notion of the ideal player, which is limited in consistency and results in repetitive misjudgments (Williams and Reilly, 2000). Such holistic models consider the players' physical, technical, and psychosocial attributes and are essential in successful talent identification and development environments, but are also likely to be a fertile area for additional research (Kelly and Williams, 2020; Vaeyens et al., 2008).

There is speculation that the influence of relative age and biological maturation could negatively impact traditional and modern approaches to talent identification and development (Smith et al., 2018). Relative age refers to the variation in chronological age within a specific age group, which is determined by comparing individuals' dates of birth and the cutoff dates for that particular age group (Hill et al., 2020). Since youth athletes are traditionally grouped by chronological age (e.g., U10, U11, U12, etc.) to provide fair competition and equal opportunity, there could be a considerable age gap of up to 12 months among participants within the same cohort. Such a scenario is even more problematic when biological maturation is neglected (Hill et al., 2020). The advancement towards the state of maturity or adulthood that is characterized by status, timing and tempo can be referred to as biological maturation (for details, see Malina et al., 2019). Large interindividual variability rates characterize biological maturation and indicate that children within the same chronological age do not mature simultaneously, with some individuals maturing in advance or delayed relative to their peers (Jackowski et al., 2011). Such considerable variability in somatic and biological growth has often been described in the literature (Hill et al., 2020), with potential consequences for variables associated with soccer performance (Kelly et al., 2021). Evidence suggests that there are significant advantages in height, lean body mass and components associated with performance in individuals with advanced maturity status (Figueiredo et al., 2009; Selmi et al., 2020; Vaeyens et al., 2006). Hence, player selection biases may occur due to relative age and biological maturation and cause profound impacts on talent identification and development programs.

Relative age and biological maturation should be acknowledged as separate constructs that exist and function independently. Such consideration might avoid potential selection biases and optimize processes for identifying and developing young talents in professional soccer academies (Hill et al., 2020). Recently, promising approaches have been developed and tested to group young soccer players based on their biological maturation instead of their chronological age (Cumming et al., 2017). These new competitive formats offer advantages for talent identification and development programs (Malina et al., 2019). However, implementing such innovative approaches in soccer is challenging since it requires appropriate methods that meet ethical and economically pragmatic criteria to assess biological maturity status (Leyhr et al., 2020). Biological maturity status in youth athletes is commonly determined using various methods, categorized into skeletal, sexual or somatic maturity indicators (Baxter-Jones et al., 2005). Despite the accuracy of methods utilized in evaluating biological maturation, their effectiveness is constrained by factors such as equipment accessibility, analysis proficiency requisites, assessment methods’ invasiveness, as well as legal and ethical concerns coupled with time and financial restraints (Lloyd et al., 2014). To overcome such barriers, at least in part, Petersen et al. (1988) developed a scale that continuously measures pubertal status and significantly correlates with other methods of biological maturation. This scale, known as Pubertal Developmental Scale (PDS), includes questions on the gonads, adrenals, and growth factors that change the body during the pubertal period (Petersen et al., 1988). The PDS has been recently introduced as a valuable tool for estimating pubertal development in soccer studies (Perroni et al., 2018, 2019, 2023). However, it is currently unknown whether the PDS-derived measures of pubertal status are significantly related, and comparable to those found for chronological age and relative age, to the physical capabilities of soccer players. Hence, the present study investigated the relationships of chronological age, relative age, and PDS-derived pubertal status measures with selected performance variables in young soccer players.

## Methods

### Participants

Sixty-one well-trained young male soccer players (mean age: 13.5 ± 0.6 years; body height: 169.0 ± 8 cm; body mass: 59.0 ± 10 kg) from amateur (n = 36) and professional (n = 26) soccer teams voluntarily participated in the study. All participants had a minimum of six years of continuous soccer training and competitive experience and played in both national and local championships. Participants had at least three training sessions per week (~90 to 120 min per session), with an official soccer match (60 min per match) at the end of the week. All participants followed a similar training program consisting of technical and tactical skill development and physical conditioning under the supervision of the same qualified physical trainer. They were free from injuries limiting their ability to train and complete the performance tests. They were accustomed to the testing procedures and were asked to abstain from strenuous exercise for at least 24 h prior to the performance tests. The study was evaluated and approved by the local research ethics committee of the University of Turin (protocol n. 134685, approval date: 15 July 2016) and carried out in accordance with the most recent revision of the Declaration of Helsinki. All participants and their parents provided their written informed consent before the study began, after being informed about the experimental procedures, potential risks, and benefits of participation.

### Design and Procedures

A descriptive correlation design was used to explore the relationship among age, biological maturity status, and variables of soccer-related performance. A convenience sample of youth soccer players was recruited, and chronological age, relative age, and PDS-derived measures of puberal status (Petersen et al., 1988) were then determined. Players’ anthropometric characteristics and physical capabilities using field tests for sprinting (10-m linear sprint test; Cronin and Templeton, 2008; Delaney et al., 2018), vertical jumps (countermovement jump test; Bosquet et al., 2009), repeated sprint ability (7 × 34.2-m shuttle sprints with a slalom; Bangsbo, 1994), and intermittent high-intensity endurance (Yo-Yo intermittent recovery test level 1; Castagna et al., 2006; Krustrup et al., 2003) were also determined. This battery of performance tests could describe the players’ neuromuscular and endurance characteristics. Performance testing was carried out at the beginning of a training season to limit the differences in training status among participants. Each testing session, conducted within the same week, spanned two days with an identical sequence. On the first day, players visited the medical room for anthropometric measurements and completed the PDS scale. They then followed a warm-up protocol (details below) and underwent anaerobic evaluations in this order: a countermovement jump, a 10-m linear sprint, and a repeated sprint ability test. To mitigate the effects of fatigue, participants received a 10 to 15-min recovery period after each test before the subsequent one. On the second day, after the same warm-up, players performed an intermittent high-intensity endurance test. All testing sessions were completed under comparable environmental conditions (temperature range: 18–22°C) and within a designated time frame (14:00–18:00), using an artificial turf that met the standards set for competitions at a national level (Stiles et al., 2009). Prior to all performance tests, participants completed a standardized warm-up, which included ten minutes of submaximal running and five minutes of athletic drills (such as skipping and high-knee runs), with three minutes of dynamic stretching in between. All participants had prior knowledge of the testing protocols due to their regular involvement in soccer club assessments.

### Measures

#### Anthropometric Evaluation

All measurements were taken in the morning by the same trained evaluator. Body height was measured with a fixed stadiometer (model 702, Seca GmbH, Germany) and body mass with a digital scale (model 813, Seca GmbH, Germany), with precision of ± 0.1 kg and ± 0.1 cm, respectively.

#### Pubertal Development

The PDS was used to measure pubertal development, as it is a valid and reliable self-assessment scale which significantly correlates with other measures of pubertal status, including physician ratings (Petersen et al., 1988). This scale comprises five questions about gonadal, adrenal, and growth factors that alter the body during puberty. For each question, participants were asked to rate changes in body height, body hair, facial hair, skin, and voice, using a 4-point scale (1 = has not begun yet, 2 = barely begun, 3 = definitely begun, 4 = seems complete). The PDS values were utilized to calculate the average PDS score (PDS_avg5_), the PDS category score (PDS_cat3_), and the pubertal category. The PDS_avg5_ score was calculated by summing the responses of all five items, divided by the number of items. Otherwise, the PDS_cat3_ score was calculated by summing the body hair growth, voice change, and facial hair items. Finally, the pubertal category, obtained from the PDS_cat3_ score, was used to categorize participants as early pubertal (score = 3, 4, or 5), mid-pubertal (score = 6 to 8), and late pubertal (score = 9 to 12) (Carskadon and Acebo, 1993). The Italian-translated version of the PDS (Perroni et al., 2014) was adopted in this study and used to relate soccer players' pubertal development and physical capabilities (Perroni et al., 2018). The questionnaire was completed individually in a conference room, with the silent presence of an investigator who helped when needed.

#### Relative Age

Chronological age was defined as the number of days since birth. Relative age, on the other hand, was defined based on age quarters. Participants were assigned to one of four relative age quartiles based on their month of birth, regardless the year of birth. Specifically, Q1 referred to individuals born between January and March, Q2 to those born between April and June, Q3 referred to individuals born between July and September, and Q4 to those born from October to December. This categorization was chosen because the cutoff date for all sports in Italy, including soccer, is January 1^st^.

#### Performance Evaluation

*Countermovement jump test*. Each participant performed a vertical jump test using an optical time system (model Optojump, Microgate, Bolzano, Italy). This system has been proposed to be valid for estimating jumping height by measuring flight time as traditional contact mats do (Bosquet et al., 2009; Castagna et al., 2013). Each participant was instructed to maintain an initial standing position with the plantar part of the foot contacting the ground and hands on the hips. Then, they performed a countermovement until the knee angle was ~90° and immediately jumped as high as possible. All players performed three barefoot countermovement jumps (CMJ) interspersed with 2 min of rest in between. The highest jump with proper execution was used for the final data analysis. The CMJ is widely used as a valid and highly reliable (test-retest stability coefficient of 0.80–0.98) performance test to assess the explosive strength of the leg musculature of athletes (Bosco et al., 1983; Slinde et al., 2008). However, to account for the differences in body mass, CMJ data were converted into “work” by multiplying force (body mass × acceleration of gravity) × distance (jump height, in m).

*Ten-meter linear sprint test*. Linear sprint performance was evaluated using a valid and reliable 10-m standing start sprint test (Cronin and Templeton, 2008; Delaney et al., 2018). Two photoelectric cells (Polifemo, Microgate, Udine, Italy) placed 0.75 m above the ground and positioned 10 m apart with the first timing gate at 0.5 m from the start were used. All participants performed three linear sprint attempts, interspersed with 5-min rest intervals. The best 10-m sprint time was used for further analysis.

*Yo-Yo intermittent recovery test*. The Yo-Yo Intermittent Recovery Test level 1 (YYIRT1) was used to assess aerobic fitness. It is recognized as a valid indicator of physical performance related to soccer matches (Krustrup et al., 2003). Previous research has reported a substantial intraclass correlation coefficient of 0.78, further affirming its reliability (Fanchini et al., 2014). The YYIRT1 was performed according to the procedures suggested by Krustup et al. (2003) and Castagna et al. (2006). All participants performed 2 × 20-m sprints, back and forth, with their speed progressively increasing between starting and finishing lines, controlled by audible bleeps. After each running bout, participants were allowed a 10-s rest interval to recover, during which they jogged around a cone placed 5 m behind the starting line. Participants experienced increasing increments of speed until they either failed twice to reach the line when the sound signal was given or were too tired to keep running at the required speed despite verbal encouragement. The measurement of the total distance covered was regarded as the outcome of the test (Krustrup et al., 2003).

*Repeated sprint ability test*. A Repeated Sprint Ability (RSA) test, consisting of seven 34.2 m shuttle sprints with a slalom on a turf surface, was performed to measure the ability to repeatedly produce maximal or near maximal short-duration sprints with brief recovery periods (Bangsbo, 1994). Each sprint was followed by 25 s of passive recovery, during which the participant jogged back to the starting line (Wragg et al. 2000). Verbal feedback was given at 5, 10, 15, and 20 s of the recovery period. Researchers instructed all participants to exert maximum effort during each sprint and avoid pacing themselves. Each sprint time was recorded to the nearest 0.01 s using a digital chronometer connected to photoelectric cells (S30-A, Digitech, Trieste, Italy) positioned at the starting and finishing lines. Both total time (RSA_TT_), which is the sum of the seven 34.2-m shuttle sprints with a slalom, and a fatigue index (RSA_FI_), a measure of the performance decline demonstrated over the entire RSA test, were recorded and used for the final data analysis (Fitzsimons et al., 1993).

### Statistical Analysis

Descriptive statistics are reported as mean ± standard deviation. The normality assumption was verified using the Shapiro-Wilk W-test. A one-way multivariate ANOVA was used to compare physical and performance variables among pubertal categories, followed by Tukey’s post-hoc tests. A correlation matrix assessed correlations between PDS-derived measures of pubertal status (PDS_avg5_, PDS_cat3_, and pubertal category). Correlation analyses were also performed to determine the relationship among chronological age, relative age, and PDS-derived measures of pubertal status with selected measures of physical performance (CMJ height, CMJ “work”, 10-m linear sprint time, YYIRT1 covered distance, and RSA_TT_ and RSA_FI_). The correlation coefficient (*r*) and the standard error of estimate (SEE) were reported as a result of each linear regression model. The level of significance was set at *p* < 0.05. All the analyses were conducted using SPSS Statistics 26.0 and RStudio.

## Results

**Figure 1:**
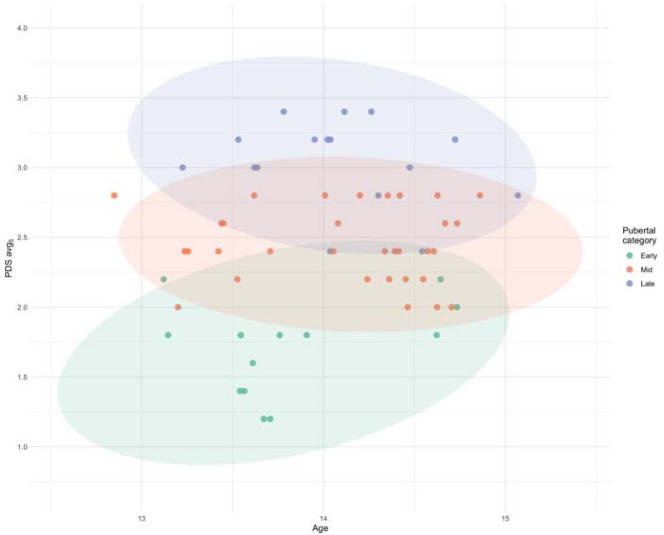
Scatter-plot of the relationship between chronological age (years) and the average PDS score (PDS_avg5_). The 95% confidence area for each pubertal category is represented by a colored ellipse (i.e., early pubertal (green, n = 13); mid-pubertal (orange, n = 32), and late pubertal (blue, n = 16)).

Sixty-one well-trained young male soccer players participated in the investigation. According to the positional roles, they were categorized as defenders (n = 25), midfielders (n = 13), strikers (n = 15), and goalkeepers (n = 8). Most participants were aged 13 (n = 25; 41%) or 14 (n = 34; 56%), with two players aged 12 and 15. Figure 1 illustrates the relationship between chronological age, expressed as the number of days since birth, and the PDS_avg5_. There was a large interindividual variability in pubertal status within the same age group in the studied population. In particular, participants were classified as early pubertal (n = 13; 21%), mid-pubertal (n = 32; 53%), and late pubertal (n = 16; 26%). Table 1 shows the descriptive data of the selected measures of physical performance, grouped by PDS-derived indices of the pubertal category. Significant differences between pubertal categories were revealed for body height (F_(2,58)_ = 12.6; *p* < 0.001), body mass (F_(2,58)_ = 11.8; *p* < 0.001) and work in the CMJ test (F_(2,58)_ = 6.0; *p* < 0.001), with early pubertal players showing lower measures in all variables.

**Table 1:** Descriptive statistics (mean ± standard deviation) of the anthropometric indicators and selected measures of soccer performance, grouped by PDS-derived indices of the pubertal category.

	Early pubertal (n = 13)	Mid pubertal (n = 32)	Late pubertal (n = 16)
Age (years)	13.2 ± 0.4	13.7 ± 0.5	13.7 ± 0.6
Body height (cm)	160.8 ± 6.3	171.5 ± 6.0 ^a^	170.6 ± 7.9 ^a^
Body mass (kg)	48.8 ± 10.3	61.5 ± 7.9 ^a^	63.0 ± 9.1 ^a^
CMJ (cm)	29.6 ± 2.9	30.2 ± 4.9	29.5 ± 3.0
CMJ “Work” (J)	142.5 ± 40.4	182.2 ± 37.3 ^a^	182.8 ± 32.1 ^a^
10-m linear sprint (s)	1.86 ± 0.08	1.84 ± 0.09	1.82 ± 0.12
YYIRT1 (m)	1255 ± 382	1174 ± 382	1214 ± 396
RSA_TT_ (s)	49.2 ± 1.6	49.7 ± 2.4	49.3 ± 2.4
RSA_FI_ (%)	4.26 ± 1.70	4.13 ± 2.42	4.25 ± 2.17

CMJ, countermovement jump; CMJ “Work”, estimated work at the countermovement jump; YYIRT1, Yo-Yo intermittent recovery test level 1; RSA, repeated sprint ability; TT; total time; FI, fatigue index. ^a^ significant difference from “Early pubertal”, p < 0.05

As expected, correlation coefficients between different PDS-derived measures of pubertal status, namely, PDS_avg5_, PDS_cat3_, and the pubertal category, were relatively high (range *r* = 0.84–0.91; *p* < 0.05). Such PDS indices, however, were scarcely related to selected measures of physical performance. Significant correlations were only found between different PDS-derived measures of pubertal status and “work” in the CMJ test (range *r* = 0.33–0.36; *p* < 0.01). Table 2 shows Pearson’s correlation coefficients between PDS-derived pubertal development indices and selected physical performance measures.

**Table 2:** Correlation and standard error of the estimate (SEE) between PDS-derived indices of pubertal development and selected measures of physical performance.

	PDS_avg5_		PDS_cat3_		Pubertal category	
	***r***	**SEE**	***r***	**SEE**	***r***	**SEE**
CMJ	−0.006	4.04	−0.013	4.04	−0.007	4.04
CMJ “Work”	0.366 †	36.95	0.351 †	37.18	0.336 †	37.39
10-m linear sprint	−0.215	0.09	−0.220	0.09	−0.153	0.09
YYIRT1	0.066	379.89	0.026	380.59	−0.031	380.54
RSA_TT_	−0.109	2.38	−0.115	2.37	0.018	2.39
RSA_FI_	0.028	2.20	−0.045	2.20	0.002	2.20

Note. CMJ, countermovement jump; CMJ “Work”, estimated work at the countermovement jump; YYIRT1, Yo-Yo intermittent recovery test level 1; RSA, repeated sprint ability; TT; total time; FI, fatigue index. † p < 0.01

Descriptive data of the selected measures of physical performance, grouped by chronological age and relative age (four quarters according to the player's birth month), are reported in Table 3. Pearson’s correlation coefficients between chronological age, relative age, and selected measures of physical performance are shown in Table 4. Similar to the PDS-derived measures of pubertal status, both chronological age and relative age were poorly related to selected measures of physical performance. A significant correlation was only found between chronological age and CMJ height (*r* = −0.297; *p* = 0.02) (Table 4).

**Table 3:** Descriptive statistics (mean ± standard deviation) of the anthropometric indicators and selected measures of physical performance, grouped by chronological age and relative age.

	Q1	Q2	Q3	Q4
***Age group, 13-yrs***				
N (count)	11	2	8	4
Body height (cm)	163.7 ± 7.8	169.5 ± 0.7	167.6 ± 10.3	170.3 ± 8.2
Body mass (kg)	52.2 ± 11.3	58.2 ± 4.8	56.2 ± 12.7	61.5 ± 13.0
CMJ (cm)	30.2 ± 2.7	29.9 ± 1.7	31.3 ± 6.9	30.3 ± 4.8
CMJ “Work” (J)	155.4 ± 42.3	170.3 ± 4.4	174.3 ± 60.6	186.4 ± 60.2
10-m linear sprint (s)	1.85 ± 0.07	1.88 ± 0.04	1.83 ± 0.12	1.81 ± 0.15
YYIRT1 (m)	1396.4 ± 347.5	1500.0 ± 28.3	1382.5 ± 405.6	895.0 ± 318.9
RSA_TT_ (s)	48.4 ± 2.2	48.1 ± 0.7	48.8 ± 2.8	50.4 ± 1.0
RSA_FI_ (%)	3.33 ± 1.84	2.91 ± 1.05	4.81 ± 2.41	5.71 ± 1.39
***Age group, 14-yrs***				
N (count)	19	7	7	1
Body height (cm)	168.8 ± 5.4	175.4 ± 5.0	173.0 ± 9.9	170.0
Body mas (kg)	59.3 ± 7.9	68.4 ± 4.1	62.9 ± 10.9	63.9
CMJ (cm)	29.3 ± 4.4	29.3 ± 1.7	29.3 ± 2.5	25.4
CMJ “Work” (J)	171.1 ± 37.0	196.2 ± 12.0	179.9 ± 26.5	159.2
10-m linear sprint (s)	1.84 ± 0.09	1.83 ± 0.06	1.82 ± 0.14	1.96
YYIRT1 (m)	1059.8 ± 369.4	1104.2 ± 384.7	1177.5 ± 308.9	1321.4
RSA_TT_ (s)	50.1 ± 2.4	50.2 ± 3.0	49.4 ± 2.4	49.9
RSA_FI_ (%)	3.99 ± 2.74	4.07 ± 1.75	4.30 ± 1.68	4.97

Q1–Q4, age quarter; CMJ, countermovement jump; CMJ “Work”, estimated work at the countermovement jump; YYIRT1, Yo-Yo intermittent recovery test level 1; RSA, repeated sprint ability; TT; total time; FI, fatigue index

**Table 4:** Correlation and standard error of the estimate (SEE) between chronological age (days since birth), relative age (year’s quartile), and selected measures of physical performance.

	Chronological age		Relative age, 13-yrs		Relative age, 14-yrs	
	***r***	**SEE**	***r***	**SEE**	***r***	**SEE**
CMJ	−0.297 †	3.86	−0.002	3.04	0.194	4.44
CMJ “Work”	0.002	39.71	0.122	31.85	0.201	49.02
10-m linear sprint	−0.089	0.09	0.032	0.10	−0.130	0.09
YYIRT1	−0.242	369.45	−0.099	341.01	0.087	376.92
RSA_TT_	0.197	2.34	0.068	2.30	−0.069	2.39
RSA_FI_	−0.068	2.19	0.333	1.72	0.190	2.68

Note. CMJ, countermovement jump; CMJ “Work”, estimated work at the countermovement jump; YYIRT1, Yo-Yo intermittent recovery test level 1; RSA, repeated sprint ability; TT; total time; FI, fatigue index. † p < 0.01

## Discussion

To the best of the authors' knowledge, this study is the first to characterize the relationship among different PDS-derived measures of pubertal status, chronological age, relative age, and selected performance variables within a group of well-trained young soccer players that was narrowed in terms of age and training status. The principal novel finding of this investigation was that physical performance, except the vertical jump, of young soccer players seemed not to be related to chronological age, relative age, or different PDS-derived measures of pubertal status. This is somewhat surprising considering the reports indicating that differences in chronological age between the youngest and oldest individuals within an age group and/or advanced maturity status within the same cohort have been linked to advantages in body height, lean body mass, and performance (Figueiredo et al., 2009; Vaeyens et al., 2006). Significant correlations were only found between different PDS-derived measures of pubertal status and the variable “work” in the CMJ test and between chronological age and CMJ height. Such findings agree with prior reports on the potential impact of chronological age and/or biological maturation on young soccer players' neuromuscular performance, particularly the vertical jump (Perroni et al., 2014; Radnor et al., 2021). It is worth noting, however, that there were more players born in the first quarter of the selection year than in any other quarter of the year. This over-representation of players born in the first part of the selection year has also been reported in other sports and is of paramount importance for the identification and development of talent.

Traditionally, youth soccer competitions have been organized into annual age groups (e.g., U10, U11, U12, etc.) in which athletes are divided into categories to ensure fair competition and equal opportunity. Consequently, there may be almost 12 months of difference between the youngest and the oldest athletes in the same cohort, where those who are born early in the selection year (e.g., first birth quarter) tend to have anthropometric and physical advantages compared with those born later in the selection year (e.g., fourth birth quarter) (Smith et al., 2018). This difference in chronological age between the youngest and oldest individuals within an age category is commonly referred to as relative age; the outcomes arising from this phenomenon are known as the relative age effect (Hill et al., 2020). The relative age effect is problematic because it leads to a skewed distribution of birth dates in many sports, with an over-representation of athletes who were born in the first half of the selection year (Helsen et al., 2000, 2005). Such over-representation suggests that traditional approaches to talent identification and development in soccer are mostly based on physical capabilities rather than technical or tactical skills (Chmura et al., 2022; Helsen et al., 2000). Nevertheless, our findings confirm the existing literature by showing that the proportion of players born in the first quarter of the selection year (50.8%) is much higher than the proportion of players born in all other quarters (49.2%). This skewed birthdate distribution towards an earlier birthdate from the age group cutoff date contrasts with the even distribution of birthdates in the Italian population. The potential causes and consequences of this overrepresentation have yet to be fully understood, though interest in this curious phenomenon has increased exponentially in recent years (Jakobsson et al., 2021).

The relative age effect has often been highlighted in soccer literature (e.g., Smith et al. (2018)), while few scientific reports have attempted to explore physical advantages purported for relatively older players (e.g., Carling et al., 2009). Surprisingly, our research findings did not support previous studies (Carling et al., 2009; Deprez et al., 2012; Hirose, 2009; Lovell et al., 2015) which indicate that soccer players who are born in the first quarter of the selection year tend to have greater body height and mass compared to those born in the fourth quarter, although the small sample size makes our data hardly generalizable (Table 3). It has been previously reported that players born in the last quarter tend to mature earlier, offsetting the relative age effect and thus competing physically with their relatively older teammates (Deprez et al., 2012, 2013). Whether differences in body size, regardless of biological maturity status, might impact selected performance variables of young soccer players remains to be elucidated. Some findings, nevertheless, indicate that when considering players at the representative level, there is limited disparity in physical abilities between individuals born within the initial and final quarters of their respective age categories (Lovell et al., 2015). The present results confirm this observation and further indicate the absence of significant correlations between chronological age, relative age, and physical capabilities in young soccer players, despite the significant correlation between chronological age and CMJ height (Table 4). These findings confirm that the relative age effect on selected performance variables may not be easily identifiable in a relatively narrow group of young soccer players regarding chronological age and training status. The findings also suggest that coaches and talent scouts demonstrate a bias towards players who possess superior physical abilities, since individuals born in the fourth quarter of the selection year tend to mature earlier (Deprez et al., 2013); this enable them to better physically compete against their comparatively older peers. This hypothesis, however, could not be tested in the context of the present study and remains a topic for future investigations.

The general assumption is that biological maturation may influence the processes of identifying and developing young talents in soccer academies (Hill et al., 2020; Zanetti et al., 2021). Biological maturation is characterized by large interindividual variability rates and indicates that children within the same chronological age do not mature simultaneously, with some individuals maturing in advance or delayed relative to their peers (Jackowski et al., 2011). However, advanced maturity status within the same cohort is associated with advantages in body size, fat-free mass, and fitness components of importance for youth soccer performance (Figueiredo et al., 2009; Vaeyens et al., 2006). Interestingly, there is no evidence of overrepresentation of early maturing players in the studied sample (see Table 1; early pubertal, 21% vs. late pubertal, 26%). This finding is similar to a previous study (Grendstad et al., 2020) and indicates that players' physical capabilities, combined with other technical, tactical, and psychosocial attributes, and not biological maturity status per se, impact the processes of selecting young talents in soccer academies. This speculation is confirmed by our study since PDS-derived measures of pubertal status were scarcely related to selected measures of physical performance (Table 2). Hence, advanced maturity status per se is not always linked to advantages in terms of physical and physiological characteristics in young soccer players. There were, however, significant relationships among chronological age, biological maturity status, and the vertical jump in the studied population (Table 2). The vertical jump was determined using a CMJ, a simple, valid, and reliable surrogate of the explosive strength of the leg musculature of athletes (Bosco et al., 1983; Slinde et al., 2008). CMJ test results, primarily adjusted by body mass, might indicate that older players or those advanced in maturity status outperform their younger or less mature counterparts (Tables 2 and 4) (Perroni et al., 2018). Whether vertical jumps are more susceptible than other physical capabilities for identifying variations in biological maturation within a relatively narrow sample of youth soccer players in terms of chronological age and training status is uncertain and warrants further investigation.

Some limitations of the present study should be mentioned. The most prominent limitation was the assessment of biological maturation (Baxter-Jones et al., 2005). Biological maturity status in youth athletes is determined using various methods, categorized into skeletal, sexual or somatic maturity indicators. Nevertheless, although these methods are deemed valid and reliable, they are constrained by factors such as the accessibility of equipment, necessity for proficiency in analysis techniques, time and cost implications, invasiveness of some measurements, as wells as legal and ethical concerns (Lloyd et al., 2014). Alternative methods for estimating biological maturity status, such as self-reported puberty status, have been recently proposed and introduced in soccer studies, but their limitations in accuracy are still debated (Malina et al., 2012, 2019). The studied convenience sample size is relatively small within each group and narrowed in terms of age and the competitive level, as can be seen in Table 3. The small sample size, particularly in some quarters, makes descriptive statistics unreliable to those groups, and therefore, generalizing players from other chronological ages and competing at different levels should be made with caution.

## Conclusions

These findings indicate that the potential effects of age and biological maturation on selected performance variables might not be easily identifiable in a group of young soccer players narrowed in terms of chronological age and training status. Biologically advanced soccer players, therefore, had no physical advantages and could equally compete physically with their respective biologically delayed teammates. Whether such findings would be related to a selection phenomenon that started before the studied age (13–14 years), however, cannot be concluded and should be a topic of future studies.
